# From industrial research to academic discoveries, toward a new concept of partnership: the Biomathics model

**DOI:** 10.3389/fphar.2014.00166

**Published:** 2014-07-28

**Authors:** Olivier Beauchet, Christine Merjagnan-Vilcoq, Cédric Annweiler

**Affiliations:** ^1^Division of Geriatric Medicine and Memory Clinic, Department of Neuroscience, Angers University Hospital and UPRES EA 4638, University of Angers, UNAMAngers, France; ^2^BiomathicsParis, France; ^3^Robarts Research Institute, The University of Western OntarioLondon, ON, Canada

**Keywords:** academic, collaboration, consortium, data basis, initiative, model, network, research

Recently, Winblad and colleagues (Lundkvist et al., 2014) reported an interesting point of view highlighting the current failures of the pharmaceutical industry to find an effective drug solution against Alzheimer's disease, and the difficulties rising from greater Research and Development costs in the context of growing regulatory and administrative straitjacket. This paradigm shift may result to a critical situation of decline of Alzheimer's research and therapy. Alzheimer's disease is a worldwide big concern because of its expanding prevalence and incidence, and its numerous adverse consequences including loss of autonomy, poor quality of life, high morbid-mortality and institutionalization (Prince et al., 2013). No curative treatment has been discovered so far, and the only treatment options are symptomatic (Anand et al., 2014). Thus, in order to reduce Alzheimer's impact and costs, the development of curative drugs proves necessary.

One solution emphasized by Winblad and colleagues to avoid this crisis is the back to basics way, meaning turning to non-profit academic research to break the deadlock (Lundkvist et al., 2014). The value of academic research is based on the small size of research teams, avoiding too much inertia and making it possible to work through close interaction rather than contracting; on the opportunity to test candidate molecules; and also on their ability to work on rare diseases and orphans drugs in the CNS area that may provide indirect solutions to the problem of Alzheimer's disease (Lundkvist et al., 2014). Eventually, most importantly, these biomedical academic research teams have the opportunity to develop more easily translational research and to conduct action-research, a way of thinking together and combining research and clinical practice (Beauchet et al., 2012).

The academic approach is indeed still promising. Nevertheless, it is worth pointing out that research as conceived hitherto could not answer a number of important issues, leaving the door open for the pharmaceutical industry. It is therefore highly suitable to make academic research evolve. The main problem was the excessive fragmentation, dispersion and confinement of skills and knowledge fiercely guarded by every academic research team. Research should overcome these difficulties and open up to others. Future of research will undoubtedly be based on the pooling of resources, research, databases, and teams. This is exactly the purpose of Biomathics consortium.

Biomathics is an emerging scientific research consortium applied to human systems modeling intended to help physicians and scientists worldwide to work together and think faster and wider. Firmly focused on the fields of human longevity, autonomy and prediction of health issues and their adverse consequences, Biomathics Consortium aims at generating initiatives, linking data from research and data from quantified-self, i.e., the self-measure of health and function using digital technologies to promote health in the general population (den Braber, 2013). Biomathics involves researchers worldwide, operates international collaborations, interconnects research databases on health and well-being, and initiates and/or joins international research programs.

Biomathics connects academic research teams working in the same research fields, and offer them to share their respective basis in order to compound a larger and more comprehensive data basis. Of course, this organization process requires to evaluate similar or close outcomes, and to adopt a shared language. Nevertheless, methods based on effect size or z-score can combine slightly different variables. This allows extremely fast answers to research questions with only little additional financial resources and using very large population-based samples. Moreover, it is also likely that some endpoints identified in a specific study may be of concern to another team, or at least may respond to queries of this second team. In such case, the requesting team launches an initiative within Biomathics and contacts all teams likely to help. Willing researchers are included in the initiative, participate in the research, revise the collaborative publication and are included in the list of co-authors based on their involvement in the work and the number of participants made available.

The main strengths of Biomathics Consortium are to make a formal link between worldwide leading research teams on aging and longevity, specifically those who have not worked together thus far. It allows building an effective worldwide operational network, applying to grants as a research network, training students and fellows, and offering immediate reactivity and high efficiency. It offers data extracted from various international clinical researches. It makes also possible to screen for potential participants for future research programs. Last, but not least, it promotes the emergence of new ideas and the validation of pilot projects through international multicentric studies. For this reason, Biomathics Consortium is a typical and distinctive model of “bottom-up” operation in the context of “top-down” reorganization of most research programs due to budgetary constraints and restrictions. While research studies are increasingly built to meet government programs and grants (“top-down” model), Biomathics Consortium is raised by researchers for themselves and is intended to explore research questions that do not necessarily receive the attention of governing bodies. In convergence with top-down organization, rapid production of big results would eventually influence the decision-making and change health and research policies, hence the “bottom-up” word acceptation.

In conclusion, we are at a crucial time for research. Times are changing, research too. Tomorrow's opportunities imply global convergence, efficient networks, and merging academic legitimacy and flexibility with private funds and stringency to keep producing innovative science not restricted by commercial objectives. Biomathics Consortium is intended to unify forces in order to improve the efficiency of research and quickly find effective treatment options for patients. Since this is still, and always must be, researchers' first priority.

## Author contributions

All authors meet all of the following criteria: (1) contributing to the conception and design, or analyzing and interpreting data; (2) drafting the article or revising it critically for important intellectual content; and (3) approving the final version to be published.

### Conflict of interest statement

The authors declare that the research was conducted in the absence of any commercial or financial relationships that could be construed as a potential conflict of interest.
